# One Cup of Coffee a Day Modulates the Relationship between Metabolic Syndrome and Eating Fast: A Cross-Sectional Study

**DOI:** 10.3390/healthcare12060603

**Published:** 2024-03-07

**Authors:** Reisa Hiramatsu, Etsuko Ozaki, Satomi Tomida, Teruhide Koyama

**Affiliations:** 1 Department of Epidemiology for Community Health and Medicine, Kyoto Prefectural University of Medicine, Kyoto 602-8566, Japan; 2 Department of Endocrine and Breast Surgery, Kyoto Prefectural University of Medicine, Kyoto 602-8566, Japan

**Keywords:** cardiometabolic diseases, metabolic syndrome, speed eating, coffee, beverage

## Abstract

Background: Eating speed has been implicated as a leading cause of metabolic syndrome (MetS). However, it is difficult to break the habit of eating fast. Since coffee consumption is associated with a lower risk of MetS, we investigated the association between eating speed and the amount of coffee consumed with MetS. Methods: This study included data from 3881 participants (2498 females and 1383 males). We used a self-administered questionnaire to collect information on participants’ coffee consumption (<1 cup/day and ≥1 cup/day) and eating speed (slow, normal, or fast). Odds ratios (ORs) and 95% confidence intervals (CIs) were calculated using logistic regression analyses to investigate the relationship between the prevalence of MetS due to eating speed and the amount of coffee consumed. Results: The group that consumed ≥1 cup/day of coffee (filtered or instant) had a lower OR for MetS compared to the group that consumed <1 cup/day (OR: 0.695; 95% Cl: 0.570–0.847). The eating fast group, compared to the eating slow group, had a higher OR for MetS (OR: 1.689; 95% Cl: 1.227–2.324). When the data were stratified by coffee consumption and eating speed groups, the slow-eating group among those who consumed <1 cup/day of coffee (filtered or instant) had a lower OR for MetS (OR: 0.502; 95% CI: 0.296–0.851) compared to the fast-eating group. In contrast, the groups who consumed ≥1 cup/day of coffee were associated with lower OR for MetS, regardless of their eating speed. Conclusions: This suggests that drinking ≥1 cup/day of coffee may help prevent MetS induced by eating fast.

## 1. Background

Metabolic syndrome (MetS) is associated with an increased risk of developing cancer [1], cardiovascular diseases [2], and other medical conditions. It is also associated with a 1.5-fold increased risk of mortality from any cause [2]. MetS is caused by an unhealthy diet, physical inactivity, excessive alcohol consumption, and work-related psychological factors; however, eating speed has also been implicated [3]. Eating slowly using a self-administered questionnaire has been shown to reduce body mass index (BMI) and waist circumference (WC) [4]. Furthermore, several studies on the relationship between eating speed and MetS using a self-administered questionnaire have found that eating slowly reduces the risk of developing MetS [3,5].

According to Health Japan 21 (the second term), approximately 15 million Japanese people are reported to have MetS (https://www.nibiohn.go.jp/eiken/kenkounippon21/en/index.html, accessed on 28 February 2024). On the other hand, coffee is one of the most popular non-alcoholic beverages worldwide. Currently, coffee is the world’s second most exported commodity after oil. Japan is the third-largest consumer of coffee beans worldwide [6]. Previous research has suggested that coffee consumption is associated with a low risk of MetS [7]. Coffee beans contain high amounts of chlorogenic acid (CGA), which is an important biologically active dietary polyphenol. The biological properties of CGA (antioxidant and anti-inflammatory effects) have recently been reported [8], suggesting that CGA may play a pivotal role in the regulation of glucose and lipid metabolism and their related disorders, such as diabetes, cardiovascular disease, obesity, cancer, and hepatic steatosis [9].

Some longitudinal studies using a self-administered questionnaire have suggested that eating fast is positively associated with MetS and an increase in BMI in the Japanese population [10,11]. Although it is difficult to break the habit [12], it may be possible to prevent MetS by drinking coffee, which is popularly consumed worldwide. This study used a cross-sectional design to investigate the association between eating speed and the amount of coffee consumed, either respectively or in combination with MetS.

## 2. Methods

### 2.1. Study Population and Design

**Figure 1:**
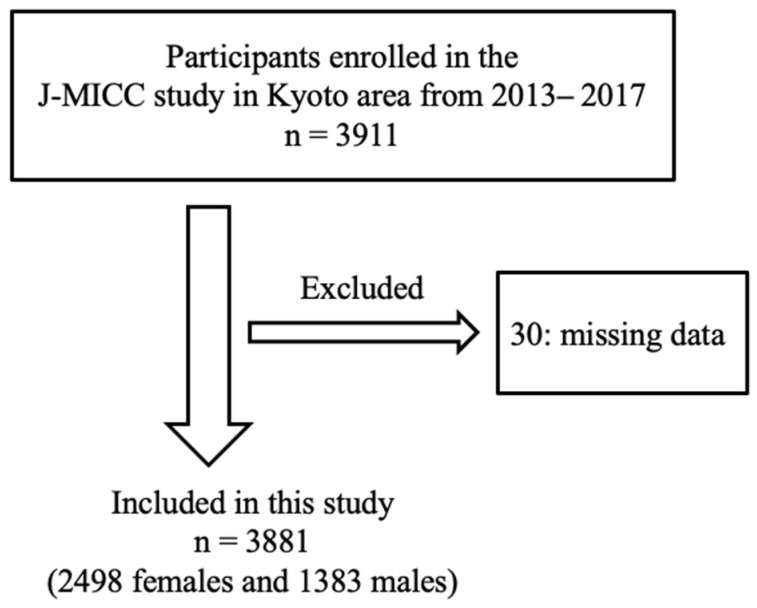
Flow chart of the study participants.

We launched a cohort study known as the Japan Multi-Institutional Collaborative Cohort Study (J-MICC study) in 2005 [13]. The current cross-sectional study included data from individuals who participated in the J-MICC study’s follow-up (2013–2017) surveys in Kyoto, Japan [14]. Of the 3911 participants, 30 were excluded due to missing data. Thus, this study’s population ultimately consisted of 3881 participants (2498 females and 1383 males). Figure 1 shows the flow chart of the study participants. All participants were recruited within the framework of the J-MICC study follow-up survey in the Kyoto area. All the participants provided written informed consent before the survey. The study protocol was approved by the Institutional Ethics Committee of the Kyoto Prefectural University of Medicine in 2013 (approval number: RBMR-E-36) and was conducted in accordance with the Declaration of Helsinki.

### 2.2. Data Collection and Measurements

All participants underwent routine health examinations, including anthropometry (weight, height, and WC), blood pressure, and venous blood sampling. We used a self-administered questionnaire to collect information on participants’ coffee consumption, eating speed (slow, normal, or fast), smoking and drinking status (never, former, or current), medication use, history of ischemic heart disease and stroke, average daily physical activity time, and type and frequency of leisure activities.

Information on the frequency of coffee consumption was obtained in the J-MICC study’s follow-up (2013–2017) surveys in Kyoto from seven choices (never, <2 cups/week, 3–4 cups/week, 5–6 cups/week, 1–2 cups/day, 3–4 cups/day, and ≥5 cups/day) for each of the following two types of coffee: “filtered or instant” and “canned, bottled or packed”. These seven choices were then separated into the following two categories: <1 cup/day and ≥1 cup/day. A validated short food-frequency questionnaire (FFQ) was used to estimate daily energy intake [15,16,17,18]. The frequency of the consumption of 46 foods and beverages within the year prior to the J-MICC study was obtained. Among the 46 foods, the frequency of staple food consumption, i.e., rice, bread, and noodles, at breakfast, lunch, and dinner, was categorized into groups, from rarely to every day. For the remaining 43 foods and beverages, the frequency of consumption was categorized into eight groups, from rarely to ≥3 times per day. The short FFQ demonstrated high reproducibility and reasonable validity for food group consumption [19].

To assess medication use, the participants were asked whether they were taking medication for hypertension, dyslipidemia, or diabetes at least once a week. Physical activity was quantified using a format similar to that of the short version of the International Physical Activity Questionnaire [20]. Physical activity was measured as metabolic equivalents (METs), as previously reported [21,22], estimated by multiplying the reported time spent daily on each activity by the corresponding metabolic equivalent intensity.

BMI was calculated as the weight divided by height in meters squared (kg/m^2^). According to the Japanese criteria, a female or male with MetS is described as having a WC of ≥90 cm or ≥85 cm, respectively, in addition to two or more of the following: lipid abnormalities (a triglyceride level ≥ 150 mg/dL, high-density lipoprotein cholesterol (HDL-C) level ≤ 40 mg/dL, or the use of lipid-modifying medications); elevated blood pressure (systolic blood pressure (SBP) ≥ 130 mmHg, diastolic blood pressure (DBP) level ≥ 85 mmHg, or the use of antihypertensive medications); and elevated blood glucose (HbA1c level ≥ 5.6% or the use of diabetes medications) [23].

### 2.3. Statistical Analysis

Continuous variables are expressed as the mean ± standard deviation, and categorical variables are expressed as sums and percentages. Intergroup comparisons were performed using Kruskal–Wallis for continuous variables and chi-squared tests for categorical variables. Kruskal–Wallis was also used for group comparisons. Table 1 shows the characteristics of the participants, stratified according to their eating speed. Odds ratios (ORs) and 95% confidence intervals (CIs) were calculated using logistic regression analyses to assess the relationship between eating speed and coffee consumption with the prevalence of MetS. The model was adjusted for age (years), sex, METs, energy (kcal), history of ischemic heart disease and stroke, drinking and smoking status (never, former, or current). Coffee consumption (“filtered or instant” and “canned, bottled or packed”), and eating speed were simultaneously used as explanatory variables. In brief, age (years), sex, METs, energy, history of ischemic heart disease and stroke, and drinking and smoking status, coffee consumption (“filtered or instant” and “canned, bottled or packed”) and eating speed are used as explanatory variables in the logistic model in Table 2. Coffee consumption of less than 1 cup/day and eating fast were used as references. In Table 3, the two coffee (filtered or instant) consumption groups (<1 cup/day and ≥1 cup/day) and the three eating speed groups (slow, normal, or fast) are stratified by dividing each predictor variable into six groups. We used coffee intake and eating speed categorized into six groups, which are included as explanatory variables in the logistic model of Table 3. The model was adjusted for age (years), sex, METs, energy (kcal), history of ischemic heart disease and stroke, and drinking and smoking status (never, former, or current). Coffee consumption of less than 1 cup/day and eating fast were used as references. We additionally performed stratified analyses by sex. Statistical significance was set at *p* ≤ 0.05. All data were analyzed using the SPSS software (version 25).

## 3. Results

The proportion of the participants in the slow-eating and fast-eating groups was 14.0% (n = 350) and 33.8% (n = 845), respectively, for females and 12.5% (n = 173) and 39.8% (n = 550), respectively, for males. A total of 15.3% (n = 595) participants had MetS. The average age was 57.5 years (standard deviation, 9.94; range, 39–75). The slow-eating group had a lower mean BMI and WC than the fast-eating group for both females and males.

Table 2 shows the association of MetS with coffee consumption (“filtered or instant” and “canned, bottled, or packed”); eating speed was also used as an explanatory variable. The group that consumed ≥1 cup/day of coffee (filtered or instant) had a lower OR for MetS compared to the group that consumed <1 cup/day of coffee (OR: 0.695; 95% Cl: 0.570–0.847; *p* < 0.001). Similar results were found when the data were analyzed by sex. By contrast, sex difference was observed in the group that consumed canned, bottled, or packed coffee; females who consumed ≥1 cup of coffee/day compared to the females who consumed <1 cup/day of coffee had a higher OR (OR: 2.056; 95% Cl: 1.110–3.811; *p* = 0.022) for MetS. The participants who consumed ≥1 cup of coffee/day (filtered or instant) had a lower OR for MetS. Regarding eating speed, eating fast compared to eating slow had a higher OR for MetS (OR: 1.689; 95% Cl: 1.227–2.324; *p* = 0.001). Similar results were found when the data were analyzed by sex.

Table 3 shows the interaction between MetS, eating speed, and coffee consumption (filtered or instant). In the slow-eating group, those who consumed <1 cup/day of coffee (filtered or instant) had a lower OR for MetS (OR:0.502; 95% CI: 0.296–0.851) compared to those in the fast-eating group. In contrast, the groups who consumed ≥1 cup/day of coffee were associated with lower OR for MetS, regardless of their eating speed. Similar results were found when the data were analyzed for females. For males, there was no significant statistical analysis, and there was a trend towards lower ORs for MetS in the slow-eating group among those who consumed <1 cup/day of coffee (filtered or instant).

## 4. Discussion

We investigated the relationship between eating speed and MetS and the effect of coffee on MetS. Participants who consumed ≥1 cup/day of coffee (filtered or instant) had a lower OR for MetS. When the data were stratified by coffee consumption and eating speed groups, the slow-eating group among those who consumed <1 cup/day of coffee (filtered or instant) had a lower OR for MetS compared to the fast-eating group. In contrast, the groups who consumed ≥1 cup/day of coffee were associated with lower OR for MetS, regardless of their eating speed. To our knowledge, the present study is the first to report no relationship between eating fast and MetS when ≥1 cup of coffee/day (filtered or instant) is consumed. In contrast, females who consumed ≥1 cup of canned, bottled, or packed coffee/day had a higher OR for MetS. The proportion of females who consumed ≥ 1 cup of coffee/day (canned, bottled, or packed) was approximately 5%, which was lower than that of males (approximately half of the male). It is not clear whether this result was due to sex differences or to a bias in the number of participants. This study targeted local residents and recruited participants by mailing recruitment invitations, so there was a difference in the number of females and males. Studies that have investigated the relationship between coffee and MetS have reported conflicting results, which could be due to the coffee variety and/or the food ingredients added to the coffee (milk, sugar, etc.) [24]. A study reported that participants who drank instant coffee mix containing sugar and powdered creamer ≥3 times/day were 1.37 times more likely to be obese than those who drank the same type of coffee <1 time/week [25]. The content of coffee, including added sugar or powdered creamer, can increase the risk of metabolic disorders [25]. Even in Japan, canned coffee, bottled coffee, and packaged coffee, such as instant coffee, often contain sugar or a powdered creamer. However, the coffee variety or presence/absence of sugar in coffee was not obtained in this study. Therefore, studies are needed to investigate the effects of sugar in coffee.

Over the years, eating speed has been an important factor in the development of obesity. Several hypotheses have been proposed to explain why eating fast can lead to MetS. First, due to a lack of satiety, eating fast may lead to overeating before the stomach feels full [26]. Second, eating fast is independently associated with insulin resistance, which is one of the major risk factors for MetS [27]. The prevalence of obesity (BMI ≥ 25 kg/m^2^) among the three eating speed groups (slow, medium, and fast) was 14.6%, 23.3%, and 34.8%, respectively, with the fast-eating group having the highest prevalence of obesity [28]. In addition, Zhu et al. examined the relationship between eating speed and the incidence rates of MetS [29]. During the 3-year follow-up period, 647 participants were diagnosed with MetS. The multivariate-adjusted hazard ratio for the incidence of MetS in the fast-eating group, compared with the slow-eating group, was 1.30 (95% CI: 1.05–1.60) after adjusting for potential confounders. Eating speed was also found to be significantly related to WC and lipid metabolic risk factors [29]. There have been many reports on the relationship between eating speed and MetS [28,30,31].

Coffee, one of the world’s most popular beverages, contains a variety of chemicals, including caffeine and polyphenols. The anti-obesity effects of coffee have also been demonstrated [32]. Most studies have focused on the beneficial effects of caffeine [33,34]. Caffeine alters the body’s energy balance by increasing energy expenditure and decreasing energy intake, potentially making it useful as a body weight regulator [35]. Coffee without caffeine also has beneficial effects in humans [36,37]. Coffee contains many polyphenols, particularly CGAs, which are believed to have antioxidant properties. CGA, compared with a placebo, has been shown in some studies to significantly reduce weight [38]. These effects are known to reduce the risk of developing obesity and MetS, and the current results suggest that coffee consumption may inhibit MetS induced by eating fast.

This study has several limitations. First, this study used a cross-sectional design; therefore, we could not observe the participants in a time series. Second, we did not assess the details of coffee consumption, such as the cup size, use of caffeinated or decaffeinated coffee, or the method of preparation. The amount of caffeine, CGA, and other ingredients effective against MetS in coffee varies with each coffee [39]. We minimized this limitation by dividing the characteristics of coffee into two groups, including “filtered or instant” and “canned, bottled or packed”. However, we could not categorize according to additives, such as sweet coffee or coffee with milk. Third, this study used subjective questionnaires to assess the speed of eating and the amount of coffee consumed. Therefore, the responses may not accurately reflect the actual eating and drinking habits of the participants. Fourth, the participants were limited to the Japanese population, so it is unclear whether a similar result would be observed in different country groups. Fifth, we often refer to BMI; however, this measures not only the amount of fat but also muscle. Sixth, we have no data on when the participants consume coffee. Therefore, we cannot discuss the effects of the timing of coffee consumption.

## 5. Conclusions

Participants who consumed ≥1 cup/day of coffee (filtered or instant) had a lower OR for MetS and eating slow compared to eating fast was associated with a lower likelihood of developing MetS. In contrast, the groups consuming ≥1 cup/day of coffee were associated with a lower OR for MetS, regardless of eating speed, compared to those consuming <1 cup/day of coffee (filtered or instant) in the fast-eating group. This suggests that drinking ≥1 cup of coffee/day (filtered or instant) may help prevent MetS induced by eating fast.

## Figures and Tables

**Table 1:** Characteristics of participants according to eating speed.

		Eating Speed						
		Slow		Normal		Fast		*p*-Value
		Mean/n	SD/%	Mean/n	SD/%	Mean/n	SD/%	
All		n = 523		n = 1963		n = 1395		
	Sex (female), n [%]	350	[66.9]	1303	[66.4]	845	[60.6]	0.001
	Age (years), mean [SD]	57.9	[10.2]	58.0	[9.9]	56.5 ^a^*	[9.8]	<0.001
	METs (h/day), mean [SD]	14.2	[10.3]	14.8	[10.4]	14.4	[10.7]	0.225
	Smokers, n [%]							
	Current	44	[8.4]	181	[9.2]	127	[9.1]	0.049
	Former	115	[22.0]	434	[22.1]	367	[26.3]	
	Never	364	[69.6]	1348	[68.7]	901	[64.6]	
	Current drinkers, n [%]							
	Current	315	[60.2]	1143	[58.2]	830	[59.5]	0.542
	Former	5	[1.0]	30	[1.5]	26	[1.9]	
	Never	203	[38.8]	791	[40.3]	539	[38.6]	
	Coffee (drip, filter, or instant) ≥ 1 cup/day, n [%]	336	[64.2]	1312	[66.8]	981	[70.3]	0.019
	Coffee (canned, plastic bottle, or carton) ≥ 1 cup/day, n [%]	39	[7.5]	157	[8.0]	127	[9.1]	0.387
	Ischemic heart disease, n [%]	3	[0.6]	18	[0.9]	3	[0.2]	0.038
	Stroke, n [%]	11	[2.1]	32	[1.6]	21	[1.5]	0.655
	BMI (kg/m^2^), mean [SD]	21.5	[3.2]	22.0 ^b^**	[3.1]	22.9 ^b^**	[3.3]	<0.001
	Waist circumference (cm), mean [SD]	79.1	[9.1]	80.2 ^a^*	[9.1]	82.3 ^b^**	[9.4]	<0.001
	Systolic blood pressure (mmHg), mean [SD]	128	[19.8]	129	[19.1]	130	[19.3]	0.249
	Diastolic blood pressure (mmHg), mean [SD]	77.6	[11.2]	79.4 ^b^**	[11.2]	79.9 ^b^**	[11.6]	0.001
	Triglyceride (mg/dL), mean [SD]	91.5	[59.1]	100.0	[76.0]	104 ^b^**	[71.6]	<0.001
	HDL-cholesterol (mg/dL), mean [SD]	72.8	[16.6]	70.6 ^b^**	[17.5]	69.1 ^b^**	[17.3]	<0.001
	LDL-cholesterol (mg/dL), mean [SD]	122	[30.8]	126 ^a^*	[30.4]	126	[30.9]	0.040
	Glucose (mg/dL), mean [SD]	90.8	[16.4]	90.2	[13.5]	91.0	[15.6]	0.752
	Hemoglobin A1c (%), mean [SD]	5.6	[0.5]	5.6	[0.4]	5.6	[0.5]	0.516
	Energy (kcal/day), mean [SD]	1607	[317]	1586	[348]	1627	[377]	0.010
	Metabolic syndrome, n [%]	64	[12.2]	280	[14.3]	251	[18.0]	0.001
Female		n = 350		n = 1303		n = 845		
	Age (years), mean [SD]	57.2	[10.2]	57.2	[9.8]	56.3	[9.8]	0.117
	METs (h/day), mean [SD]	14.6	[10.4]	15.0	[10.3]	14.8	[10.1]	0.646
	Smokers, n [%]							
	Current	16	[4.6]	59	[4.5]	28	[3.3]	0.042
	Former	39	[11.1]	125	[9.6]	115	[13.6]	
	Never	295	[84.3]	1119	[85.9]	702	[83.1]	
	Current drinkers, n [%]							
	Current	178	[50.9]	630	[48.0]	430	[50.9]	0.754
	Former	4	[1.1]	16	[1.2]	12	[1.4]	
	Never	168	[45.8]	657	[50.4]	403	[47.7]	
	Coffee (drip, filter, or instant) ≥ 1 cup/day, n [%]	227	[64.9]	893	[68.5]	612	[72.4]	0.024
	Coffee (canned, plastic bottle, or carton) ≥ 1 cup/day, n [%]	19	[5.4]	71	[5.4]	38	[4.5]	0.597
	Ischemic heart disease, n [%]	0	[0]	5	[0.4]	0	[0]	0.101
	Stroke, n [%]	5	[1.4]	20	[1.5]	10	[1.2]	0.794
	BMI (kg/m^2^), mean [SD]	21.0	[3.2]	21.3 ^a^*	[3.0]	22.1 ^b^**	[3.3]	<0.001
	Waist circumference (cm), mean [SD]	77.2	[9.2]	78.0	[8.7]	80.0 ^b^**	[9.2]	<0.001
	Systolic blood pressure (mmHg), mean [SD]	125	[19.4]	126	[18.5]	126	[19.7]	0.378
	Diastolic blood pressure (mmHg), mean [SD]	75.6	[11.0]	77.2 ^a^*	[10.8]	77.6 ^a^*	[11.3]	0.016
	Triglyceride (mg/dL), mean [SD]	81.7	[41.3]	87.7	[64.3]	90.5 ^a^*	[50.6]	0.010
	HDL-cholesterol (mg/dL), mean [SD]	76.7	[15.8]	75.1	[16.7]	74.1 ^a^*	[16.3]	0.023
	LDL-cholesterol (mg/dL), mean [SD]	126	[31.2]	128	[30.8]	128	[32.7]	0.314
	Glucose (mg/dL), mean [SD]	88.8	[14.5]	87.9	[11.5]	88.4	[12.5]	0.986
	Hemoglobin A1c (%), mean [SD]	5.6	[0.5]	5.5	[0.4]	5.5	[0.4]	0.749
	Energy (kcal/day), mean [SD]	1502	[240]	1464	[261]	1482	[287]	0.063
	Metabolic syndrome, n [%]	21	[6.0]	74	[5.7]	70	[8.3]	0.053
Male		n = 173		n = 660		n = 550		
	Age (years), mean [SD]	59.5	[10.0]	59.7	[9.9]	56.7 ^b^**	[9.9]	<0.001
	METs (h/day), mean [SD]	13.4	[10.1]	14.2	[10.5]	13.8	[11.7]	0.361
	Smokers, n [%]							
	Current	28	[16.2]	122	[18.5]	99	[18.0]	0.790
	Former	76	[43.9]	309	[46.8]	252	[45.8]	
	Never	69	[39.9]	229	[34.7]	199	[36.2]	
	Current drinkers, n [%]							
	Current	137	[79.2]	512	[77.6]	400	[72.7]	0.158
	Former	1	[0.6]	14	[2.1]	14	[2.5]	
	Never	35	[20.2]	134	[20.3]	136	[24.7]	
	Coffee (drip, filter, or instant) ≥ 1 cup/day, n [%]	109	[63]	419	[63.5]	369	[67.1]	0.366
	Coffee (canned, plastic bottle, or carton) ≥ 1 cup/day, n [%]	20	[11.6]	86	[13.0]	89	[16.2]	0.173
	Ischemic heart disease, n [%]	3	[1.7]	13	[2.0]	3	[0.5]	0.096
	Stroke, n [%]	6	[3.5]	12	[1.8]	11	[2.0]	0.395
	BMI (kg/m^2^), mean [SD]	22.7	[2.8]	23.3 ^a^*	[2.9]	24.0 ^b^**	[3.0]	<0.001
	Waist circumference (cm), mean [SD]	83.0	[7.6]	84.5	[8.2]	85.9 ^b^**	[8.6]	<0.001
	Systolic blood pressure (mmHg), mean [SD]	135	[18.6]	136	[18.5]	135	[18.0]	0.325
	Diastolic blood pressure (mmHg), mean [SD]	81.8	[10.6]	83.8	[10.7]	83.4	[11.2]	0.107
	Triglyceride (mg/dL), mean [SD]	111	[81.0]	125	[89.9]	125	[86.6]	0.083
	HDL-cholesterol (mg/dL), mean [SD]	64.8	[15.2]	61.6 ^a^*	[15.6]	61.3 ^a^*	[15.9]	0.010
	LDL-cholesterol (mg/dL), mean [SD]	115	[28.6]	122 ^a^*	[29.1]	124 ^b^**	[29.7]	0.006
	Glucose (mg/dL), mean [SD]	94.9	[19.1]	94.9	[15.8]	94.9	[18.7]	0.244
	Hemoglobin A1c (%), mean [SD]	5.7	[0.6]	5.6	[0.5]	5.6	[0.6]	0.543
	Energy (kcal/day), mean [SD]	1817	[348]	1828	[372]	1848	[392]	0.574
	Metabolic syndrome, n [%]	43	[24.9]	206	[31.2]	181	[32.9]	0.136

Data are presented as the mean ± SD (standard deviation) or number (percentage); post hoc analysis: ^a^* <0.05 vs. slow, ^b^** <0.01 vs. slow; METs, metabolic equivalents; BMI, body mass index; HDL-cholesterol, high-density lipoprotein cholesterol; and LDL-cholesterol, low-density lipoprotein cholesterol.

**Table 2:** The relationship between metabolic syndrome, coffee, and eating speed.

		OR	95% CI	*p*-Value
All	Coffee (filtered or instant) < 1 cup/day	Ref		
	Coffee (filtered or instant) ≥ 1 cup/day	0.695	0.570–0.847	<0.001
	Coffee (canned, bottled or packed) < 1 cup/day	Ref		
	Coffee (canned, bottled or packed) ≥ 1 cup/day	1.187	0.867–1.626	0.284
	Eating speed			
	Slow	Ref		
	Normal	1.212	0.887–1.656	0.228
	Fast	1.689	1.227–2.324	0.001
Female	Coffee (filtered or instant) < 1 cup/day	Ref		
	Coffee (filtered or instant) ≥ 1 cup/day	0.570	0.410–0.792	0.001
	Coffee (canned, bottled or packed) < 1 cup/day	Ref		
	Coffee (canned, bottled or packed) ≥ 1 cup/day	2.056	1.110–3.811	0.022
	Eating speed			
	Slow	Ref		
	Normal	0.973	0.585–1.619	0.917
	Fast	1.607	0.960–2.691	0.071
Male	Coffee (filtered or instant) <1 cup/day	Ref		
	Coffee (filtered or instant) ≥1 cup/day	0.784	0.613–1.003	0.053
	Coffee (canned, bottled or packed) <1 cup/day	Ref		
	Coffee (canned, bottled or packed) ≥1 cup/day	1.030	0.722–1.468	0.872
	Eating speed			
	Slow	Ref		
	Normal	1.337	0.930–2.038	0.110
	Fast	1.750	1.171–2.616	0.006

OR, odds ratio; CI, confidence interval; Ref, reference. Adjusted for age, sex, METs, drinking and smoking status, energy (kcal), ischemic heart disease, and stroke.

**Table 3:** The interaction of metabolic syndrome with eating speed and coffee consumption.

				Number	Number of MetS	OR	95% CI	*p*-Value
All	Coffee (filtered or instant) < 1 cup/day	Eating speed	Slow	163	24	0.502	0.296–0.851	0.010
			Normal	534	117	0.734	0.525–1.028	0.072
			Fast	324	90	Ref		
	Coffee (filtered or instant) ≥ 1 cup/day	Eating speed	Slow	296	40	0.448	0.289–0.693	<0.001
			Normal	1149	163	0.482	0.353–0.658	<0.001
			Fast	820	161	0.684	0.499–0.936	0.018
Female	Coffee (filtered or instant) < 1 cup/day	Eating speed	Slow	115	8	0.414	0.182–0.942	0.036
			Normal	380	30	0.470	0.274–0.806	0.006
			Fast	201	32	Ref		
	Coffee (filtered or instant) ≥ 1 cup/day	Eating speed	Slow	214	13	0.360	0.182–0.714	0.003
			Normal	849	44	0.310	0.190–0.507	<0.001
			Fast	574	38	0.423	0.255–0.702	0.001
Male	Coffee (filtered or instant) < 1 cup/day	Eating speed	Slow	48	16	0.576	0.295–1.122	0.105
			Normal	154	87	0.963	0.631–1.469	0.860
			Fast	123	58	Ref		
	Coffee (filtered or instant) ≥ 1 cup/day	Eating speed	Slow	82	27	0.527	0.303–0.915	0.023
			Normal	300	119	0.643	0.434–0.953	0.028
			Fast	246	123	0.916	0.618–1.358	0.662

OR, odds ratio; CI, confidence interval; Ref, reference. Adjusted for age, sex, METs, drinking and smoking status, energy (kcal), ischemic heart disease, and stroke.

## Data Availability

Data are available on request from the authors.
